# Identifying Active Ingredients of the Association Between Neighborhood Disadvantage and Disordered Eating in Youth

**DOI:** 10.1002/eat.24441

**Published:** 2025-04-11

**Authors:** Megan E. Mikhail, Kelly L. Klump, Amber L. Pearson, S. Alexandra Burt

**Affiliations:** ^1^ Department of Psychology Michigan State University East Lansing Michigan USA; ^2^ Department of Psychiatry and Behavioral Sciences University of California, San Francisco San Francisco California USA; ^3^ Department of Public Health Michigan State University Flint Michigan USA; ^4^ Department of Public Health University of Otago, Wellington Wellington New Zealand

**Keywords:** childhood, disadvantage, disordered eating, neighborhood, socioeconomic status, violence

## Abstract

**Background:**

Emerging research suggests neighborhood disadvantage is associated with disordered eating (DE; e.g., binge eating, body dissatisfaction) beginning in middle childhood, with effects that increment those of proximal disadvantage (e.g., family income). Understanding factors that contribute to early risk for youth living in disadvantaged neighborhoods is critical because childhood DE predisposes youth to more severe eating disorders in adolescence/adulthood. However, the specific “active ingredients” through which neighborhoods impact DE remain unknown. We examined community violence exposure and limited neighborhood resources (e.g., green spaces, recreational facilities) as two notable neighborhood‐level stressors that could contribute to early associations between more distal disadvantage and DE.

**Method:**

Participants included girls and boys oversampled for neighborhood disadvantage from the MSU Twin Registry (*N* = 2060; *M*_age_ = 8.02; 48.7% female). Analyses used structural equation modeling to examine whether community violence exposure and neighbor informant reports of neighborhood resources were associated with DE after accounting for proximal youth characteristics (e.g., family income, racial identity).

**Results:**

Community violence exposure was significantly associated with DE (*b* = 0.20, 95% CI [0.14, 0.27]), but neighborhood resources were not (*b* = 0.04, 95% CI [−0.13, 0.22]). Associations were consistent across multiple potential moderators, including age, sex, racial identity, family income, and body mass index.

**Conclusions:**

Community violence is a significant stressor that may help explain associations between neighborhood disadvantage and DE in youth. Additional research is needed to understand the underlying cognitive, affective, and biological mechanisms through which violence exposure may increase the risk for DE in under‐resourced contexts.

**Summary**

- Neighborhood poverty is associated with disordered eating (DE) beginning in middle childhood. Understanding factors that contribute to this early elevation in risk is critical, yet the “active ingredients” through which neighborhoods impact DE remain unknown.
- We found that community violence exposure was associated with increased DE in middle childhood, but neighborhood resources were not.
- Results highlight the importance of contextual stressors in DE risk.

## 1 Introduction

Youth living in high‐poverty neighborhoods are at increased risk for a wide range of mental health concerns, including anxiety/depression (Choi et al. 2021; Xue et al. 2005), externalizing disorders (Pearson et al. 2022), and social difficulties (DeCarlo Santiago, DeCarlo Santiago et al. 2011). While neighborhood disadvantage is correlated with family income, associations tend to be moderate (*r*'s ~ 0.3 to 0.5; Hackman et al. 2012; Roubinov et al. 2018), and neighborhood poverty shows significant impacts on mental health above and beyond family‐level factors (Alexandra Burt 2014). A distinct influence of the neighborhood context is consistent with Bronfenbrenner's bioecological model of human development, which posits that environmental factors at different levels of proximity exert unique effects on behavior and wellbeing (Bronfenbrenner 1977). Investigation at both proximal and more distal levels is therefore necessary to fully understand the impacts of disadvantage on mental health.

Accumulating evidence suggests that *proximal* socioeconomic disadvantage is associated with increased risk for eating disorders (EDs; e.g., anorexia nervosa [AN], bulimia nervosa [BN], binge‐eating disorder [BED]) and disordered eating symptoms (DE; e.g., binge eating, body dissatisfaction) (Burnette, Burt, and Klump 2024; McGowan et al. 2022; Mitchison et al. 2014; Mulders‐Jones et al. 2017). In particular, food insecurity is significantly associated with DE in both adults (Hazzard et al. 2020) and youth (Bidopia et al. 2023). Much less research has examined relationships between more *distal* forms of disadvantage and DE. However, the few studies conducted thus far are consistent with the broader mental health literature in suggesting potentially unique links between neighborhood disadvantage and DE that increment the effects of proximal disadvantage (Carroll et al. 2024; Mikhail et al. 2021, Mikhail, Ackerman, et al. 2023; Simone et al. 2022). Notably, associations between neighborhood poverty and DE may emerge early, with youth from disadvantaged neighborhoods already reporting elevated symptoms in middle childhood (i.e., ages 6–11) even when controlling for family income (Carroll et al. 2024). Moreover, preliminary research suggests neighborhood disadvantage may lead to earlier activation of genetic influences on DE in pre/early puberty (Mikhail et al. 2021; Mikhail, Carroll, et al. 2023), potentially shaping developing neural circuits in ways that potentiate risk long term.

While preliminary research suggests an incremental effect of neighborhood disadvantage on DE, relatively few studies have been conducted overall, and almost nothing is known about *why* living in a disadvantaged neighborhood may lead to increased DE. While research has begun to identify mechanisms that may contribute to associations between proximal disadvantage and DE (e.g., food insecurity), no studies have yet investigated more distal neighborhood factors that may contribute to risk. Identifying the “active ingredients” through which neighborhoods may impact DE is critical to fully understand why disadvantaged youth are at increased risk and to identify potential targets for prevention.

Neighborhood disadvantage is associated with multiple factors that are linked to psychopathology in youth and may act as active ingredients to increase DE. Disadvantaged neighborhoods are less likely to contain structural resources, including green spaces, community centers, and recreational facilities, that help support healthy youth development (Henry et al. 2014; Pearson et al. 2022; Timonen et al. 2021; Zhang et al. 2020). Positive youth development theory suggests the presence of supportive resources in the community (“community assets”) is important to facilitate healthy socioemotional development in young people (e.g., social competencies, positive identity, sources of support and connection, and a sense of personal self‐efficacy; Shek et al. 2019). Conversely, youth living in neighborhoods with a dearth of structural resources are at increased risk for several negative outcomes related to DE, including anxiety/depression (Li et al. 2021), being diagnosed with a psychiatric condition (Shen 2022), decreased well‐being (Liu et al. 2018), and poorer metabolic outcomes (e.g., poorer diabetes control; Bergmann et al. 2022). Limited neighborhood resources could impact DE through mechanisms shared with other mental health concerns (e.g., increased negative affect, decreased social support, lower overall self‐esteem) as well as mechanisms more specific to DE (e.g., fewer safe areas for movement and recreation contributing to lower body esteem). However, no research has yet examined associations between structural neighborhood resources and DE in youth.

Simultaneously, individuals living in disadvantaged neighborhoods may be more likely to experience significant stressors, among the most salient of which is community violence. Youth living in disadvantaged urban and rural neighborhoods in the United States (US) are substantially more likely to witness community violence than their peers in advantaged neighborhoods (Kravitz‐Wirtz et al. 2022; Lee et al. 2003). Direct violence exposure (Brady 2008) and trauma more generally (Brewerton 2015; Trottier and MacDonald 2017; Zelkowitz et al. 2021) are associated with increased ED risk. Violence in the broader community has been far less studied, but emerging evidence suggests it may also contribute to DE. In the two studies that have examined this question explicitly, community violence exposure (i.e., witnessing or directly experiencing at least one community violence event in the past year, such as being shot or shot at or getting beaten up or mugged) was associated with DE in female and male adolescents cross‐sectionally (Isaksson et al. 2023) and across a one‐year follow‐up period (Isaksson et al. 2024). Associations between community violence exposure and DE may be mediated by increased trauma symptoms relevant to DE, including dissociation from one's body and emotion regulation difficulties (Isaksson et al. 2024; Lev‐Ari et al. 2021). However, no research has yet examined the association between community violence exposure and DE in younger populations or for youth living in disadvantaged neighborhoods where violence may occur more often. Because the two studies conducted thus far have used dichotomous measures of violence exposure (i.e., any exposure versus none), it is also unclear whether the frequency of violence exposure may linearly increase risk.

The goal of the current study was therefore to better understand the more distal active ingredients of neighborhood disadvantage that may contribute to early DE. Specifically, we examined whether (limited) neighborhood resources and/or community violence exposure were associated with DE in a large sample of girls and boys in middle childhood enriched for neighborhood disadvantage. We incorporated a deep phenotyping of neighborhood characteristics, including independent neighbor informant reports of neighborhood resources and a well‐validated measure of community violence exposure. Our large sample size and inclusion of both girls and boys (based on sex recorded on original birth certificates; hereafter referred to as sex) also allowed us to examine potential moderators of neighborhood effects. Notably, this is the first study to identify more distal factors that may contribute to DE for youth living in disadvantaged neighborhoods.

## 2 Method

### 2.1 Participants

Analyses included 2060 participants (48.7% girls, 51.3% boys) nested in 1030 families recruited through the Michigan State University Twin Registry (MSUTR; Burt and Klump 2013, 2019; Klump and Burt 2006) for the *Twin Study of Behavioral and Emotional Development in Children* (TBED‐C; Burt and Klump 2019). Prior work in this sample (Carroll et al. 2024) and the MSUTR more broadly (Mikhail et al. 2021; Mikhail, Ackerman, et al. 2023) has found associations between global neighborhood disadvantage and DE, but none have examined the impact of specific neighborhood processes on DE.

The TBED‐C consists of a population‐based arm representative of Michigan (51.3% of the sample) and an under‐resourced arm drawn from Census block groups in which ≥ 10.5% of residents had incomes at or below the federal poverty line (48.7% of the sample). Participants primarily ranged in age from 6 to 10 (*M*_age_ = 8.02, SD = 1.49), although a few pairs had turned 11 by the time they participated. Additional demographic information is included in Table 1.

**TABLE 1:** Participant descriptive statistics.

Participant characteristics	Mean (SD) or *N* (% of sample)
Sex	
Female	1003 (48.7%)
Male	1057 (51.3%)
Age	8.02 (1.49) [range = 6–11]
Zygosity	
Female twins from same sex monozygotic pairs	404 (19.6%)
Female twins from same sex dizygotic pairs	412 (20.0%)
Male twins from same sex monozygotic pairs	448 (21.7%)
Male twins from same sex dizygotic pairs	422 (20.5%)
Twins from opposite sex dizygotic pairs	374 (18.2%)
Race/ethnicity	
White (non‐Latinx)	1682 (81.7%)
Black/African American	196 (9.5%)
Latinx/Hispanic	16 (0.8%)
Asian American	16 (0.8%)
Native American/American Indian	22 (1.1%)
Pacific Islander	6 (0.3%)
Other/Unknown	122 (5.9%)
Combined parental income	
Less than $10,000	54 (2.6%)
$10,000–$15,000	74 (3.6%)
$15,000–$20,000	72 (3.5%)
$20,000–$25,000	74 (3.6%)
$25,000–$30,000	110 (5.3%)
$30,000–$40,000	166 (8.1%)
$40,000–$50,000	278 (13.5%)
Over $50,000	1184 (57.5%)
Unknown	48 (2.3%)
Mother's education level	
Less than high school	40 (1.9%)
High school graduate	134 (6.5%)
Trade school	88 (4.3%)
Some college	464 (22.5%)
Associate's degree	284 (13.8%)
Bachelor's degree	610 (29.6%)
Advanced graduate degree (e.g., master's, PhD, MD)	344 (16.7%)
Unknown	96 (4.7%)
Father's education level	
Less than high school	106 (5.1%)
High school graduate	394 (19.1%)
Trade school	100 (4.9%)
Some college	470 (22.8%)
Associate's degree	210 (10.2%)
Bachelor's degree	468 (22.7%)
Advanced graduate degree (e.g., master's, PhD, MD)	260 (12.6%)
Unknown	52 (2.5%)
BMI percentile	59.27 (29.09)
(Possible range = 0–100; data available for 98.1% of participants)	[range = 0–99.9]
Pubertal status	1.18 (0.46)
(Possible range = 1–4; data available for 95.4% of participants)	[range = 1–4]
Global neighborhood disadvantage percentile relative to all Census block groups in Michigan	57.24 (22.67)
(Possible range = 1–99; data available for 97.8% of participants)	[range = 2–99]
Exposure to community violence	3.07 (3.52)
(Possible range = 0–54; data available for 97.3% of participants)	[range = 0–33]
Neighborhood resources	10.48 (1.47)
(Possible range = 0–13; data available for 73.5% of participants)	[range = 5–13]
MEBS total score	5.49 (4.20)
(Possible range = 0–30; data available for 96.3% of participants)	[range = 0–22]

Abbreviations: BMI, body mass index; MEBS, Minnesota eating behavior survey.

Participants were assessed between 2008 and 2012. This timeframe was particularly informative for the examination of disadvantage processes because it overlapped with the Great Recession, which significantly impacted Michigan communities (Burgard et al. 2012). Children provided informed assent, and parents provided informed consent for themselves and their children. Neighbor informants provided informed consent.

### 2.2 Measures

#### 2.2.1 DE

DE was assessed using the Minnesota Eating Behavior Survey (MEBS) total score (von Ranson et al. 2005). The MEBS is a 30‐item self‐report questionnaire that assesses several domains of DE, including weight preoccupation, body dissatisfaction, compensatory behavior, and binge eating. Primary analyses focused on the total score because this overall measure has the best psychometric properties across sex and child/adolescent development (Culbert et al. 2014, 2017; Klump et al. 2007; von Ranson et al. 2005) and captures the full spectrum of DE symptoms. However, results were consistent across individual subscales in supplemental analyses (see Table S1).

The MEBS total score has adequate internal consistency in both girls and boys from age six into emerging adulthood (*α*'s > 0.77 in past research; *α* = 0.80 in the current sample) (Culbert et al. 2014, 2017; Klump et al. 2007; von Ranson et al. 2005), and its factor structure is consistent across sex (Luo et al. 2016). Internal consistency was similar in White youth (*α* = 0.80) and youth of color (*α* = 0.78) in the current sample, and we found evidence for strong measurement invariance across White youth and youth of color in supplemental analyses (see Supporting Information). The MEBS is strongly correlated with other DE measures (e.g., *r*'s ≥ 0.77 with the Eating Disorder Examination Questionnaire [Fairburn and Beglin 1994]; Klump et al. 2012) and discriminates between girls with and without an ED diagnosis (von Ranson et al. 2005). As in past research that has used the MEBS with young children (Culbert et al. 2017), research assistants read items aloud to participants aged six and seven to ensure adequate comprehension.

The MEBS (previously known as the Minnesota Eating Disorder Inventory [M‐EDI]) was adapted and reproduced by special permission of Psychological Assessment Resources, 16204 North Florida Avenue, Lutz, Florida 33549, from the Eating Disorder Inventory (collectively, EDI and EDI‐2) by Garner, Olmstead, Polivy, Copyright 1983 by Psychological Assessment Resources. Further reproduction of the MEBS is prohibited without prior permission from Psychological Assessment Resources.

#### 2.2.2 Neighborhood Resources

Neighborhood resource availability was assessed using informant reports provided by neighbors living in each family's Census tract. Census tracts are small formal geographic units containing 1200–8000 people (US Census Bureau 2020). In 2010, there were 2813 Census tracts in Michigan (US Census Bureau 2021), with more Census tracts in more densely populated areas (e.g., cities).

To obtain neighbor reports, mailings were sent to 10 randomly chosen households within a family's Census tract, with one adult at each address invited to complete the questionnaire (see Burt et al. 2020). There were a total of 1880 independent neighbor reports (mean = 4.49 per Census tract, SD = 1.63, range = 1–10) within Census tracts containing 1514 youth (73.5% of the sample). Neighbor informants were 63.2% women with a mean age of 52.61 years (SD = 15.52). Overall, neighbor informants primarily identified as White (80.6%); however, the racial demographics of neighbor informants tended to reflect those of youth participants living in the same Census tract (e.g., 52.3% of neighbor informants for Black youth participants identified as Black, while only 5.8% of neighbor informants for White youth participants identified as Black).

Neighbor informants reported on neighborhood resources using the 13‐item Resource Availability subscale of the Neighborhood Matters Scale questionnaire (NMS; Henry et al. 2014). Prior research has found the kinds of built neighborhood characteristics assessed by the NMS are associated with other behavioral health concerns (e.g., conduct problems) in youth (Burt et al. 2020; Pearson et al. 2022). The Resource Availability subscale assesses the presence/absence of observable material resources in the community, including medical services, recreational and religious facilities, public transportation, grocery stores, and green spaces (i.e., parks).

We conducted random effects models to examine whether neighbor reports in the same Census tract were significantly more similar than expected by chance. A significant proportion of the variance could be attributed to the Census tract (19.1% of variance; 95% CI = 0.15–0.24). Following guidance from the scale developers (Henry et al. 2014), we did not calculate internal consistency for the Resource Availability subscale because the presence of one resource in a neighborhood does not necessarily guarantee the presence of others. However, Henry et al. (2014) found the Resource Availability subscale items form a single factor, indicating they capture a unitary underlying construct.

#### 2.2.3 Community Violence Exposure

Community violence exposure was measured using the 27‐item KID‐SAVE (Flowers et al. 2000). Consistent with author recommendations (Flowers et al. 2000), the KID‐SAVE was administered via clinical interview to ensure comprehension in younger children. The KID‐SAVE assesses the frequency at which youth are exposed to violence in their neighborhood, including indirect violence (e.g., “I have heard about drive‐by shootings in my neighborhood”), direct violence (e.g., “someone has pulled a gun on me”), and physical/verbal abuse (e.g., “someone has threatened to beat me up”). To ensure a comprehensive assessment of community violence exposure, the current study used the KID‐SAVE total score capturing frequency of exposure to all forms of violence in one's neighborhood. While the KID‐SAVE also assesses the perceived impact of violence exposure, we focused on frequency because we hypothesized violence exposure may have a deleterious impact even if not perceived as overtly upsetting in the moment. This is also consistent with the limited prior research on community violence exposure and DE, which has focused on the occurrence of exposure rather than its perceived impact (Isaksson et al. 2023). Nevertheless, frequency and impact scores were highly correlated (*r* = 0.81), and results were identical if impact scores were used instead (see Table S2). Past research has shown good internal consistency (*α* = 0.91), test–retest reliability (*r* = 0.86 over 3 weeks), and construct validity (i.e., significant correlations with posttraumatic stress symptoms) of the KID‐SAVE frequency total score in youth (Flowers et al. 2000). Internal consistency in the current sample was adequate (*α* = 0.77).

#### 2.2.4 Covariates and Moderators

We initially conducted models with family income, racial identity, sex, age, pubertal status, and body mass index (BMI) percentile as covariates to identify unique effects of neighborhood processes above and beyond these more proximal factors. We then examined all covariates except pubertal status as moderators to identify potential differences in the effects of neighborhood factors across these variables. Pubertal status was not examined as a moderator because most participants were in pre/early puberty, limiting power to detect moderation (see Table 1).

Youth's parents reported their annual family income as < $10,000, $10,000–$15,000, $15,000–$20,000, $20,000–25,000, $25,000–$30,000, $30,000–$40,000, $40,000–$50,000, or > $50,000. Family income was modeled continuously as a covariate (coded from 0 = <$10,000 to 7 = > $50,000) and categorically in moderation analyses (≤$50,000 or > $50,000) to allow for sufficient observations at each level of the moderator.

Racial identity was assessed via questionnaire based on US Census categories and coded as White, Black/African American, Latino/a/x, Asian/Asian American, Native American, or other race/ethnicity (recoded as White, Black/African American, or other race/ethnicity for moderation analyses to ensure sufficient observations in each group).

Age was included continuously as a covariate and dichotomized as 6–8 or 9–11 years old when modeled as a moderator. Pubertal status was measured using the Pubertal Development Scale (PDS; Petersen et al. 1988). Although most participants were in pre/early puberty, we covaried pubertal status in addition to age to account for potential acceleration of pubertal development in disadvantaged contexts (Acker et al. 2023).

Finally, age‐ and sex‐specific BMI percentiles were calculated from height and weight measured by trained research assistants. Moderation analyses examined differences in the impact of neighborhood factors for participants above and below the median BMI percentile in the sample.

### 2.3 Statistical Analyses

In the case of missing data on individual scales, raw scores were prorated if ≤ 10% of items were missing using the formula $\begin{array}{c} \left(\frac{\text{total score across nonmissing items}}{\text{number of nonmissing items}}\right) \end{array}$ × (number of items in full scale) and marked as missing otherwise. We first examined bivariate correlations between variables to provide an initial indication of associations. We then used structural equation modeling to examine associations between DE and neighborhood resources and community violence exposure while accounting for covariates (see Figure S1). Structural equation models were conducted in Mplus version 8 (Muthén and Muthén 1998–2011) with robust full information maximum likelihood estimation (FIML) to account for missing data (Enders and Bandalos 2001) and the “complex” option to account for clustering of twins within families. FIML produces relatively unbiased estimates with even 50% missing data, although standard errors tend to be overestimated as missingness increases (leading to conservative estimates) (Schlomer et al. 2010). Statistical significance was determined using the percentile bootstrapping method with 1000 random samples with replacement (Falk 2018; Preacher and Hayes 2008); paths were deemed significant if the 95% confidence interval did not contain zero.

We then examined whether youth characteristics moderated associations between neighborhood characteristics and DE. Moderation analyses tested whether associations between neighborhood resources and/or community violence exposure and DE could be constrained to equality across levels of the moderator without worsening model fit. The constrained model was preferred if the change in chi‐square was non‐significant and Akaike's Information Criterion (AIC), Bayesian Information Criterion (BIC), and sample‐size adjusted BIC (SABIC) were lower for the constrained model relative to the full model. If AIC, BIC, and SABIC identified different models as best‐fitting, the model that optimized two out of three fit indices was selected.

Finally, we conducted exploratory analyses to investigate whether there was a significant indirect effect from global neighborhood disadvantage to DE through community violence exposure (see Figure S2). These analyses should be interpreted with caution given the limitations of cross‐sectional mediation (e.g., inability to infer directionality; O'Laughlin et al. 2018) but can inform future longitudinal research. A significant indirect effect would suggest the relationship between global neighborhood disadvantage and DE can be attributed in part to greater levels of community violence in more disadvantaged neighborhoods. As in past research (Carroll et al. 2024; Mikhail et al. 2021; Mikhail, Carroll, et al. 2023), global neighborhood disadvantage was calculated based on the Area Deprivation Index (Singh 2003). This measure incorporates 17 socioeconomic indicators (e.g., unemployment rate, median home value) assessed at the Census block group level (i.e., the smallest United States Census Bureau geographic unit). Authors calculated scores using publicly available data from the American Community Survey for the Census block group containing each family's address, with each item standardized before calculating the total score.

## 3 Results

Participants varied considerably in DE (range = 0–22, possible range = 0–30), neighborhood resources (range = 5–13, possible range = 0–13), and community violence exposure (range = 0–33, possible range = 0–54). Correlations between family income and community violence exposure (*r* = −0.16; 2.6% of variance shared), neighborhood resources (*r* = 0.05; 0.3% of variance shared), and global neighborhood disadvantage (*r* = −0.40; 16.0% of variance shared) were small to moderate, indicating significant variance unique to more distal neighborhood factors (see Table 2).

**TABLE 2:** Bivariate correlations between study variables.

	DE	Nbhd disad.	Violence	Resources	Age	Puberty	Income	BMI	Sex	Black	Latino	Asian	Native	Other
DE	—													
Nbhd Disad.	**0.13**	—												
Violence	**0.20**	**0.20**	—											
Resources	−0.03	**−0.09**	**−0.13**	—										
Age	**−0.05**	**−0.08**	**0.06**	−0.01	—									
Puberty	0.04	−0.002	**0.06**	−0.03	**0.40**	—								
Income	**−0.12**	**−0.40**	**−0.16**	**0.05**	**0.05**	**−0.05**	—							
BMI	**0.18**	**0.09**	**0.06**	0.01	**0.09**	**0.19**	**−0.07**	—						
Sex	−0.04	−0.03	**−0.12**	−0.01	0.03	**0.35**	0.01	−0.01	—					
Black	**0.15**	**0.33**	**0.21**	**−0.17**	−0.04	**0.11**	**−0.32**	**0.08**	−0.02	—				
Latino	0.02	−0.004	0.003	0.03	**0.04**	0.03	0.03	0.01	0.002	—	—			
Asian	0.01	−0.02	−0.01	0.03	0.04	**0.05**	0.004	0.01	0.02	—	—	—		
Native	0.02	0.03	−0.01	−0.01	**−0.06**	−0.01	**−0.10**	0.02	−0.01	—	—	—	—	
Other	**0.05**	**0.09**	0.02	0.05	−0.03	−0.004	**−0.11**	−0.01	−0.01	—	—	—	—	—

*Note*: Sex is coded such that the reference group is male. Bolded values are statistically significant at *p* < 0.05.

Abbreviations: BMI, body mass index percentile; DE, Minnesota Eating Behavior Survey total score; income, family income; Native, Native American racial identity; Nbhd Disad, global neighborhood disadvantage; Other, other racial identity; puberty, Pubertal Development Scale score; Resources, neighborhood resources; Violence, exposure to community violence.

At a bivariate level, DE was significantly correlated with community violence exposure (*r* = 0.20, *p* < 0.001) but not neighborhood resources (*r* = −0.03, *p* = 0.268). Results were similar when accounting for covariates in the full structural equation model. DE was significantly associated with community violence exposure (unstandardized *b* = 0.20, 95% CI [0.14, 0.27]), indicating a unique association between community violence exposure and DE after accounting for proximal disadvantage and youth demographic characteristics (see Table 3). However, DE was not significantly associated with neighborhood resources (unstandardized *b* = 0.04, 95% CI [−0.13, 0.22]).

**TABLE 3:** Associations between neighborhood factors and disordered eating with covariates included in the model.

Parameter	Standardized estimate	Unstandardized estimate	Unstandardized SE	Unstandardized 95% CI
Community violence exposure	**0.17**	**0.20**	**0.03**	**[0.14, 0.27]**
Neighborhood resources	0.01	0.04	0.09	[−0.13, 0.22]
Age	**−0.08**	**−0.21**	**0.07**	**[−0.35, −0.08]**
Pubertal status	0.01	0.13	0.29	[−0.39, 0.71]
Sex	−0.02	−0.15	0.21	[−0.56, 0.25]
Racial identity				
Black	**0.09**	**1.31**	**0.46**	**[0.41, 2.20]**
Latino	0.02	1.00	1.29	[−1.84, 3.46]
Asian	0.01	0.54	1.08	[−1.36, 2.95]
Native American	0.01	0.44	0.99	[−1.52, 2.28]
Other	0.04	0.70	0.42	[−0.14, 1.49]
Family income	−0.04	−0.09	0.06	[−0.20, 0.04]
BMI percentile	**0.17**	**0.02**	**0.004**	**[0.02, 0.03]**

*Note*: All dimensional variables (community violence exposure, neighborhood resources, age, pubertal status, family income, and BMI percentile) are modeled continuously. Sex is coded such that the reference group is male. Statistically significant paths are bolded. While the negative association between age and disordered eating in this model was somewhat unexpected, a similar small decrease in disordered eating across middle childhood has also been observed in past research (Davison, Markey, and Birch 2003; Evans et al. 2013; Knez et al. 2006). Bolded values are statistically significant at *p* < 0.05.

Abbreviation: BMI, body mass index.

### 3.1 Moderation of Associations Between Neighborhood Factors and DE

For all potential moderators, the model that constrained associations between community violence exposure and neighborhood resources and DE to equality across levels of the moderator demonstrated superior fit on at least two out of three fit indices, with no significant change in chi‐square compared to the full model (see Table 4). This indicated that associations between neighborhood factors and DE were similar across the variables examined.

**TABLE 4:** Model fit comparisons constraining associations between neighborhood factors and disordered eating across subgroups.

Model	AIC	BIC	SABIC	χ^2^ Δ (df)	*p*
Tests of moderation by sex					
Full model with moderation of community violence exposure and neighborhood resources	50702.53	51716.02	51144.14	—	—
No sex difference in association between community violence exposure and DE	50703.43	51711.28	51142.59	1.82 (1)	0.177
No sex difference in association between neighborhood resources and DE	50701.08	51708.93	51140.24	0.52 (1)	0.470
No sex difference in associations between both community violence exposure and neighborhood resources and DE	**50702.40**	**51704.63**	**51139.11**	**2.94 (2)**	**0.230**
Tests of moderation by age (6–8 or 9–11)					
Full model with moderation of community violence exposure and neighborhood resources	50573.33	51744.47	51083.63	—	—
No age difference in association between community violence exposure and DE	50571.34	51736.84	51079.18	0.002 (1)	0.961
No age difference in association between neighborhood resources and DE	50571.37	51736.87	51079.22	0.03 (1)	0.856
No age difference in associations between both community violence exposure and neighborhood resources and DE	**50569.37**	**51729.24**	**51074.77**	**0.03 (2)**	**0.985**
Tests of moderation by family income (Under or Over $50,000 annually)					
Full model with moderation of community violence exposure and neighborhood resources	44841.78	45851.02	45279.15	—	—
No difference in association between community violence exposure and DE across income	44843.43	45847.06	45278.37	2.69 (1)	0.101
No difference in association between neighborhood resources and DE across income	44840.08	45843.71	45275.02	0.25 (1)	0.617
No difference in associations between both community violence exposure and neighborhood resources and DE across income	**44841.53**	**45839.56**	**45274.04**	**2.87 (2)**	**0.238**
Tests of Moderation by BMI (Under or Over Median BMI in the Sample)					
Full model with moderation of community violence exposure and neighborhood resources	52135.18	53302.34	52641.51	—	—
No difference in association between community violence exposure and DE across BMI	52133.61	53295.16	52637.51	0.27 (1)	0.607
No difference in association between neighborhood resources and DE across BMI	52133.21	53294.76	52637.11	0.03 (1)	0.866
No difference in associations between both community violence exposure and neighborhood resources and DE across BMI	**52131.62**	**53287.56**	**52633.08**	**0.32 (2)**	**0.854**
Tests of moderation by race (White, Black, or other racial identity)					
Full model with moderation of community violence exposure and neighborhood resources	66541.36	67284.58	66865.20	—	—
No difference in association between community violence exposure and DE across race	66540.95	67272.91	66859.89	3.07 (2)	0.215
No difference in association between neighborhood resources and DE across race	66542.66	67274.62	66861.60	6.36 (2)	0.042
No difference in associations between both community violence exposure and neighborhood resources and DE across race	**66541.01**	**67261.71**	**66855.04**	**7.36 (4)**	**0.118**
Tests of moderation across population‐based and under‐resourced study arms					
Full model with moderation of community violence exposure and neighborhood resources	54475.92	55647.06	54986.23	—	—
No difference in association between community violence exposure and DE across study arms	54476.91	55642.41	54984.76	1.74 (1)	0.188
No difference in association between neighborhood resources and DE across study arms	54475.38	55640.89	54983.23	1.26 (1)	0.262
No difference in associations between both community violence exposure and neighborhood resources and DE across study arms	**54476.54**	**55636.42**	**54981.94**	**3.22 (2)**	**0.200**

*Note*: The model constraining parameters across sex removed sex as a predictor (as the model was estimated separately in males and females) and the model constraining parameters across family income removed income as a predictor (as there was no variability in the high income group given that questionnaires did not differentiate between family incomes >$50,000). The best‐fitting model is bolded. Bolded values are statistically significant at *p* < 0.05.

Abbreviations: χ^2^ Δ, Change in chi‐square; AIC, Akaike Information Criterion; BIC, Bayesian Information Criterion; DE, Minnesota Eating Behavior Survey total score; df, degrees of freedom; SABIC, Sample size adjusted Bayesian Information Criterion.

### 3.2 Exploratory Analyses of Indirect Effects From Global Neighborhood Disadvantage to DE Through Neighborhood Factors

As expected, greater global neighborhood disadvantage was associated with significantly greater community violence exposure (unstandardized *b* = 0.31, 95% CI [0.23, 0.39]; see Table S3). There was also a significant total effect from global neighborhood disadvantage to DE after accounting for covariates (unstandardized *b* = 0.13, 95% CI [0.03, 0.23]). Finally, we found a significant indirect effect from global neighborhood disadvantage to DE through community violence exposure (unstandardized *b* = 0.06, 95% CI [0.04, 0.09]), suggesting youth in disadvantaged neighborhoods may experience greater DE in part because they experience more community violence.

## 4 Discussion

This was the first study to examine “active ingredients” of neighborhood disadvantage that may contribute to DE. We focused on youth in middle childhood because prior research suggests the association between neighborhood disadvantage and DE emerges early in development (Carroll et al. 2024; Mikhail et al. 2021; Mikhail, Ackerman, et al. 2023) and identifying early risk factors may be critical for preventing later EDs (Rohde, Stice, and Marti 2015). Greater community violence exposure was associated with significantly greater DE in the full sample, with similar effects across multiple potential moderators (e.g., age, sex, racial identity). Community violence exposure was associated with DE independent of proximal disadvantage (i.e., family income) and other established correlates of DE symptoms (e.g., BMI, pubertal status). Conversely, neighborhood resources were not associated with DE in childhood. Results suggest the substantial stress associated with community violence exposure may play an important role in the relationship between neighborhood disadvantage and early DE in youth.

The significant association between community violence exposure and DE observed in this study is consistent with the well‐established relationship between trauma and EDs (Brady 2008; Brewerton 2015; Trottier and MacDonald 2017) and the limited prior research on community violence and DE specifically (Isaksson et al. 2023; Isaksson et al. 2024). Youth may become more reactive to internal sensations with frequent violence exposure, particularly normative physiological fluctuations reminiscent of fight/flight sensations, which could promote DE and EDs (Zucker and Bulik 2020). The unpredictable and uncontrollable nature of community violence may also contribute to increased negative emotions, difficulties with emotion regulation, and internalizing symptoms (Doom et al. 2024), which may in turn increase reliance on maladaptive emotion regulation strategies such as DE. To preliminarily explore this possibility, we tested whether there was a significant indirect effect from violence exposure to DE through internalizing symptoms as measured by the Semi‐structured Clinical Interview for Children and Adolescents internalizing scale (McConaughy and Achenbach 1994; see Table S4 and Figure S3). While the indirect effect through internalizing symptoms was significant, the direct effect from violence exposure to DE also remained significant. These analyses should be interpreted with caution given their cross‐sectional nature, but suggest that both internalizing symptoms and other mechanisms may contribute to community violence effects on DE (e.g., trauma symptoms, which we were unfortunately unable to test in the current sample).

Somewhat surprisingly, neighborhood resources were not significantly associated with DE. One possible explanation is that the effects of early deprivation may not become fully evident until later in development. Indeed, other studies have found delayed effects of neighborhood deprivation on cognitive (Elías Alvarado 2016; Sampson et al. 2008) and health‐related (Jimenez et al. 2019) outcomes. Research also suggests socioeconomic deprivation often has a cumulative effect, meaning the impact of reduced resources early in life can become compounded over time (Seabrook and Avison 2012). It would therefore be informative for future longitudinal studies to examine whether limited neighborhood resources in childhood have a delayed effect on DE in later adolescence/adulthood. It is also possible that social, rather than structural, neighborhood resources may have the greatest impact on DE. High neighborhood social cohesion (defined by trust, reciprocal support, and social bonds) is associated with reduced psychological distress (Rios et al. 2012) and appears to buffer the negative effects of neighborhood poverty on anxiety and depression (Dawson et al. 2019; Fone et al. 2014). Lower levels of community support may conversely increase susceptibility to negative emotions and dysregulated eating (Cortés‐García et al. 2022; Linville et al. 2012), particularly in under‐resourced contexts where youth are exposed to frequent stressors.

While we did not find moderation of disadvantage effects by race/ethnicity, Black youth lived in substantially more disadvantaged neighborhoods than White youth on average based on our measure of global neighborhood deprivation (*d* = 1.28, *p* < 0.001) and were exposed to significantly higher levels of community violence (*d* = 0.76, *p* < 0.001). Black youth are therefore disproportionately likely to be impacted by neighborhood disadvantage even if associations between neighborhood characteristics and DE are similar across race/ethnicity. Interestingly, Black youth in this sample also had higher DE even after accounting for other factors (see Table 3), potentially reflecting the additive impact of racial marginalization above and beyond effects of socioeconomic disadvantage. More research is needed on the intersectional impact of having multiple marginalized identities on DE for both Black youth and youth with other minoritized racial identities (e.g., Latino youth) whose experiences we were less able to examine in detail in the current study due to smaller sample sizes.

This study had several strengths, including a large sample of boys and girls enriched for neighborhood disadvantage, independent neighbor informant reports of neighborhood resources, and the ability to simultaneously model multiple neighborhood processes. However, some limitations should also be noted. This study was cross‐sectional; longitudinal research is needed to definitively establish community violence exposure as a risk factor for DE and understand the impact of early disadvantage on later EDs. We found initial evidence of measurement invariance for the MEBS across White youth and youth of color, but did not have sufficient power to examine invariance for specific racial groups, and comparisons across racial identity should therefore be interpreted with some caution. Youth reported their frequency of community violence exposure via interview, while neighborhood resources were reported by neighbor informants. This method variance could have contributed to the stronger association between community violence exposure and DE, particularly as differences in demographic characteristics between youth and neighbor informants (e.g., racial identity) could have contributed to differences in perceived resources. However, community violence exposure was significantly correlated with global neighborhood disadvantage (*r* = 0.20, *p* < 0.001; see Table 2), suggesting reports were tied to objective neighborhood characteristics rather than simply reflecting youth characteristics. This study did not measure potential key mechanisms of the proximal disadvantage effect such as food insecurity, and it is important for future research to examine how distal and proximal forms of disadvantage may interact in greater depth (e.g., whether the effects of community violence exposure may be greater for youth who are also experiencing food insecurity).

Findings nevertheless have potential implications for assessment and treatment of DE in disadvantaged youth. Assessment for youth with EDs should include questions about violence exposure and its potential cognitive (e.g., beliefs about self and others), emotional (e.g., negative emotional states), and physiological (e.g., increased physiological reactivity) sequelae. Prior research suggests negative beliefs regarding emotions, self, and others following traumas may contribute to DE symptoms and could be helpful to target in therapy (Trottier and MacDonald 2017). More broadly, treatment approaches should be sensitive to the impact of potentially traumatic experiences in under‐resourced youth. Lack of trauma‐informed care may lead to poorer outcomes (Rodríguez et al. 2005) and even iatrogenic effects (Brewerton 2019) in disadvantaged populations. Ongoing research is needed to continue to clarify the mechanisms contributing to symptoms and to tailor treatments to meet the unique needs of socioeconomically disadvantaged youth.

## Author Contributions

**Megan E. Mikhail:** conceptualization, formal analysis, investigation, methodology, visualization, writing – original draft. **Kelly L. Klump:** conceptualization, funding acquisition, investigation, methodology, supervision, writing – review and editing. **Amber L. Pearson:** conceptualization, funding acquisition, investigation, methodology, writing – review and editing. **S. Alexandra Burt:** conceptualization, funding acquisition, investigation, methodology, supervision, writing – review and editing.

## Ethics Statement

Study procedures were approved by the Michigan State University Institutional Review Board.

## Conflicts of Interest

The authors declare no conflicts of interest.

## Supporting information


**Data S1.** Supporting Information.

## Data Availability

The data that support the findings of this study are available from the corresponding author upon reasonable request.
