# Supplementary material for: Identifying Active Ingredients of the Association Between Neighborhood Disadvantage and Disordered Eating in Youth
Source: Int J Eat Disord. 2025 Apr 11;58(7):1307–18. doi: 10.1002/eat.24441 (PMC12227294; doi:10.1002/eat.24441)
Supplement: Supplementary file 1 — Data S1. Supporting Information. [file EAT-58-1307-s001.docx]

**Supplemental Material**

**Tests of Measurement Invariance for the Minnesota Eating Behavior Survey (MEBS)**

We conducted supplemental analyses to examine whether the MEBS showed measurement invariance across White youth (*n* = 1,682) and youth of color (*n* = 378) in the current sample. We combined youth of color (i.e., Black/African American, Latinx/Hispanic, Asian American, Native American/American Indian, Pacific Islander, and youth of other/unknown racial identity) for these analyses to ensure sufficient sample sizes in each group. Analyses used confirmatory factor analysis with WLSMV estimation given the binary nature of MEBS items and the “complex” option to account for clustering of twins within families.

A test of configural invariance using the original four subscale MEBS structure (von Ranson et al., 2005) showed adequate model fit in the current sample (RMSEA = .031, 95% CI [.028, .033], CFI = .914, SRMR = .093). Factor loadings for all individual items were significant and greater than 0.40 on their respective subscales for both White youth and youth of color. When comparing the scalar invariance (i.e., strong invariance) model to the configural invariance model, there was no significant change in chi-square (Δχ^2^(19) = 15.87, *p* = .666) or meaningful change in absolute fit indices (RMSEA = .030, 95% CI [.027, .032], CFI = .918, SRMR = .094). Thus, it is reasonable to infer strong measurement invariance for the MEBS across White youth and youth of color in the current sample.

**Table S1.** *Associations between neighborhood factors and individual MEBS subscales*

| **Parameter** | **Standardized Estimate** | **Unstandardized Estimate** | **Unstandardized SE** | **Unstandardized 95% CI** |
| --- | --- | --- | --- | --- |
| **Body Dissatisfaction** | | | | |
|  | | | | |
| **Community violence exposure** | **.08** | **.02** | **.01** | **[.01, .04]** |
| Neighborhood resources | .02 | .02 | .02 | [-.02, .06] |
| **Age** | **-.08** | **-.05** | **.02** | **[-.09, -.02]** |
| **Pubertal status** | **.07** | **.17** | **.08** | **[.04, .33]** |
| Sex | -.02 | -.05 | .05 | [-.15, .04] |
| Racial identity |  |  |  |  |
| *Black* | .04 | .14 | .13 | [-.09, .40] |
| *Latino* | .04 | .44 | .36 | [-.29, 1.12] |
| *Asian* | -.01 | -.15 | .32 | [-.67, .54] |
| ***Native American*** | **.05** | **.53** | **.26** | **[.01, 1.02]** |
| *Other* | .03 | .15 | .10 | [-.04, .35] |
| **Family income** | **-.08** | **-.05** | **.02** | **[-.08, -.02]** |
| **BMI percentile** | **.21** | **.01** | **.001** | **[.01, .01]** |
|  | | | | |
| **Weight Preoccupation** | | | | |
|  | | | | |
| **Community violence exposure** | **.12** | **.07** | **.02** | **[.03, .09]** |
| Neighborhood resources | -.02 | -.02 | .04 | [-.10, .06] |
| Age | .03 | .04 | .03 | [-.03, .11] |
| Pubertal status | .04 | .17 | .14 | [-.10, .45] |
| Sex | -.001 | -.01 | .10 | [-.21, .18] |
| Racial identity |  |  |  |  |
| ***Black*** | **.06** | **.42** | **.20** | **[.03, .82]** |
| *Latino* | -.004 | -.10 | .59 | [-1.27, 1.03] |
| *Asian* | .01 | .24 | .54 | [-.67, 1.52] |
| *Native American* | .01 | .11 | .45 | [-.69, .99] |
| *Other* | .04 | .30 | .21 | [-.10, .70] |
| Family income | -.03 | -.03 | .03 | [-.08, .02] |
| **BMI percentile** | **.18** | **.01** | **.002** | **[.01, .02]** |
|  | | | | |
| **Binge Eating** | | | | |
|  | | | | |
| **Community violence exposure** | **.16** | **.07** | **.01** | **[.05, .10]** |
| Neighborhood resources | .04 | .05 | .03 | [-.01, .10] |
| **Age** | **-.10** | **-.11** | **.03** | **[-.16, -.06]** |
| Pubertal status | -.05 | -.17 | .10 | [-.35, .03] |
| Sex | -.03 | -.09 | .08 | [-.25, .06] |
| Racial identity |  |  |  |  |
| *Black* | .02 | .10 | .16 | [-.22, .40] |
| *Latino* | .03 | .49 | .56 | [-.68, 1.51] |
| *Asian* | .01 | .21 | .48 | [-.71, 1.22] |
| *Native American* | -.01 | -.08 | .33 | [-.73, .54] |
| *Other* | .003 | .02 | .16 | [-.31, .34] |
| Family income | .004 | .004 | .02 | [-.04, .05] |
| BMI percentile | .01 | .001 | .001 | [-.002, .003] |
|  | | | | |
| **Compensatory Behavior** | | | | |
|  | | | | |
| **Community violence exposure** | **.10** | **.02** | **.01** | **[.01, .03]** |
| Neighborhood resources | .03 | .01 | .01 | [-.01, .04] |
| **Age** | **-.13** | **-.06** | **.01** | **[-.08, -.04]** |
| Pubertal status | .001 | .002 | .04 | [-.07, .07] |
| Sex | -.01 | -.01 | .04 | [-.09, .06] |
| Racial identity |  |  |  |  |
| ***Black*** | **.19** | **.46** | **.10** | **[.26, .64]** |
| *Latino* | .01 | .09 | .22 | [-.24, .53] |
| *Asian* | -.002 | -.02 | .15 | [-.26, .30] |
| *Native American* | -.01 | -.04 | .12 | [-.27, .23] |
| *Other* | .03 | .09 | .06 | [-.03, .22] |
| Family income | -.03 | -.01 | .01 | [-.03, .01] |
| BMI percentile | .02 | <.001 | .001 | [-.001, .002] |

***Note*.** MEBS = Minnesota Eating Behavior Survey; BMI = body mass index. Sex is coded such that the reference group is male. Statistically significant paths are bolded.

**Table S2.** *Associations between neighborhood factors and disordered eating, using impact rather than frequency of community violence exposure*

| **Parameter** | **Standardized Estimate** | **Unstandardized Estimate** | **Unstandardized SE** | **Unstandardized 95% CI** |
| --- | --- | --- | --- | --- |
| **Community violence exposure** | **.19** | **.23** | **.03** | **[.17, .30]** |
| Neighborhood resources | .01 | .03 | .09 | [-.13, .21] |
| **Age** | **-.07** | **-.19** | **.07** | **[-.33, -.06]** |
| Pubertal status | .02 | .14 | .29 | [-.39, .72] |
| Sex | -.03 | -.23 | .21 | [-.65, .17] |
| Racial identity |  |  |  |  |
| ***Black*** | **.09** | **1.32** | **.46** | **[.42, 2.20]** |
| *Latino* | .02 | .76 | 1.23 | [-1.95, 3.03] |
| *Asian* | .01 | .40 | 1.07 | [-1.51, 2.73] |
| *Native American* | .01 | .42 | .97 | [-1.51, 2.21] |
| *Other* | .04 | .67 | .41 | [-.17, 1.46] |
| Income | -.04 | -.08 | .06 | [-.19, .05] |
| **BMI percentile** | **.17** | **.03** | **.003** | **[.02, .03]** |

***Note*.** BMI = body mass index percentile. Sex is coded such that the reference group is male. Statistically significant paths are bolded.

**Table S3.** *Indirect effects from global neighborhood disadvantage to disordered eating through specific neighborhood factors*

| **Parameter** | **Standardized Estimate** | **Unstandardized Estimate** | **SE** | **95% CI** |
| --- | --- | --- | --- | --- |
| *Direct effects of neighborhood factors on DE* |  |  |  |  |
| **Violence exposure 🡪 DE** | **.17** | **.20** | **.03** | **[.14, .26]** |
| Neighborhood resources 🡪 DE | .01 | .04 | .09 | [-.12, .23] |
| Global neighborhood disadvantage 🡪 DE | .04 | .07 | .05 | [-.03, .17] |
|  |  |  |  |  |
| *Direct effects of global neighborhood disadvantage on specific neighborhood characteristics* |  |  |  |  |
| **Global neighborhood disadvantage 🡪**  **Violence exposure** | **.20** | **.31** | **.04** | **[.23, .39]** |
| **Global neighborhood disadvantage 🡪**  **Neighborhood resources** | **-.09** | **-.06** | **.02** | **[-.10, -.01]** |
|  |  |  |  |  |
| *Direct effects of covariates on DE* |  |  |  |  |
| **Age 🡪 DE** | **-.07** | **-.21** | **.07** | **[-.35, -.07]** |
| Puberty 🡪 DE | .02 | .14 | .29 | [-.43, .70] |
| Sex 🡪 DE | -.02 | -.15 | .21 | [-.53, .23] |
| **Black racial identity 🡪 DE** | **.08** | **1.18** | **.46** | **[.34, 2.15]** |
| Latino racial identity 🡪 DE | .02 | .99 | 1.24 | [-1.49, 3.33] |
| Asian racial identity 🡪 DE | .01 | .54 | .97 | [-1.25, 2.59] |
| Native American racial identity 🡪 DE | .01 | .43 | .99 | [-1.47, 2.46] |
| Other racial identity 🡪 DE | .04 | .65 | .42 | [-.17, 1.43] |
| Income 🡪 DE | -.03 | -.07 | .07 | [-.20, .05] |
| **BMI percentile 🡪 DE** | **.16** | **.02** | **.004** | **[.02, .03]** |
|  |  |  |  |  |
| *Indirect effects of global neighborhood disadvantage on DE* |  |  |  |  |
| **Global neighborhood disadvantage** **🡪**  **Violence exposure 🡪 DE** | **.03** | **.06** | **—** | **[.04, .09]** |
| Global neighborhood disadvantage 🡪  Neighborhood resources 🡪 DE | -.001 | -.002 | — | [-.01, .01] |
|  |  |  |  |  |
| ***Total effect of global neighborhood disadvantage on DE*** | **.07** | **.13** | — | **[.03, .23]** |

***Note*.** DE = Minnesota Eating Behavior Survey total score; violence exposure = exposure to community violence; income = family income; BMI = body mass index percentile. Sex is coded such that the reference group is male. Statistically significant paths are bolded.

**Table S4.** *Indirect effects from neighborhood factors to disordered eating through internalizing symptoms*

| **Parameter** | **Standardized Estimate** | **Unstandardized Estimate** | **SE** | **95% CI** |
| --- | --- | --- | --- | --- |
| *Direct effects of neighborhood factors and internalizing on DE* |  |  |  |  |
| **Violence exposure 🡪 DE** | **.14** | **.16** | **.03** | **[.10, .23]** |
| Neighborhood resources 🡪 DE | .01 | .03 | .09 | [-.13, .21] |
| **Internalizing 🡪 DE** | **.16** | **.15** | **.02** | **[.11, .19]** |
|  |  |  |  |  |
| *Direct effects of the neighborhood factors on internalizing* |  |  |  |  |
| **Violence exposure 🡪 Internalizing** | **.19** | **.25** | **.03** | **[.18, .31]** |
| Neighborhood resources 🡪 Internalizing | .02 | .05 | .09 | [-.11, .22] |
|  |  |  |  |  |
| *Direct effects of covariates on DE* |  |  |  |  |
| **Age 🡪 DE** | **-.08** | **-.23** | **.07** | **[-.37, -.10]** |
| Puberty 🡪 DE | .01 | .10 | .29 | [-.42, .66] |
| Sex 🡪 DE | -.03 | -.28 | .21 | [-.68, .11] |
| **Black racial identity 🡪 DE** | **.10** | **1.45** | **.45** | **[.54, 2.30]** |
| Latino racial identity 🡪 DE | .02 | .91 | 1.30 | [-2.00, 3.35] |
| Asian racial identity 🡪 DE | .004 | .17 | 1.01 | [-1.63, 2.38] |
| Native American racial identity 🡪 DE | .01 | .25 | .95 | [-1.64, 2.10] |
| Other racial identity 🡪 DE | .04 | .75 | .41 | [-.09, 1.55] |
| Income 🡪 DE | -.04 | -.10 | .06 | [-.20, .04] |
| **BMI percentile 🡪 DE** | **.17** | **.02** | **.003** | **[.02, .03]** |
|  |  |  |  |  |
| *Indirect effects of neighborhood factors on DE* |  |  |  |  |
| **Violence exposure 🡪 Internalizing 🡪 DE** | **.03** | **.04** | **—** | **[.02, .05]** |
| Neighborhood resources 🡪 Internalizing 🡪  DE | .002 | .01 | — | [-.02, .03] |
|  |  |  |  |  |
| *Total effect of neighborhood factors on DE* |  |  |  |  |
| **Violence exposure 🡪 DE** | **.17** | **.20** | — | **[.14, .26]** |
| Neighborhood resources 🡪 DE | .01 | .04 | — | [-.12, .22] |

***Note*.** DE = Minnesota Eating Behavior Survey total score; violence exposure = exposure to community violence; income = family income; BMI = body mass index percentile; internalizing = internalizing symptoms as measured by the Structured Clinical Interview for Children and Adolescents internalizing scale. Sex is coded such that the reference group is male. Statistically significant paths are bolded.

**
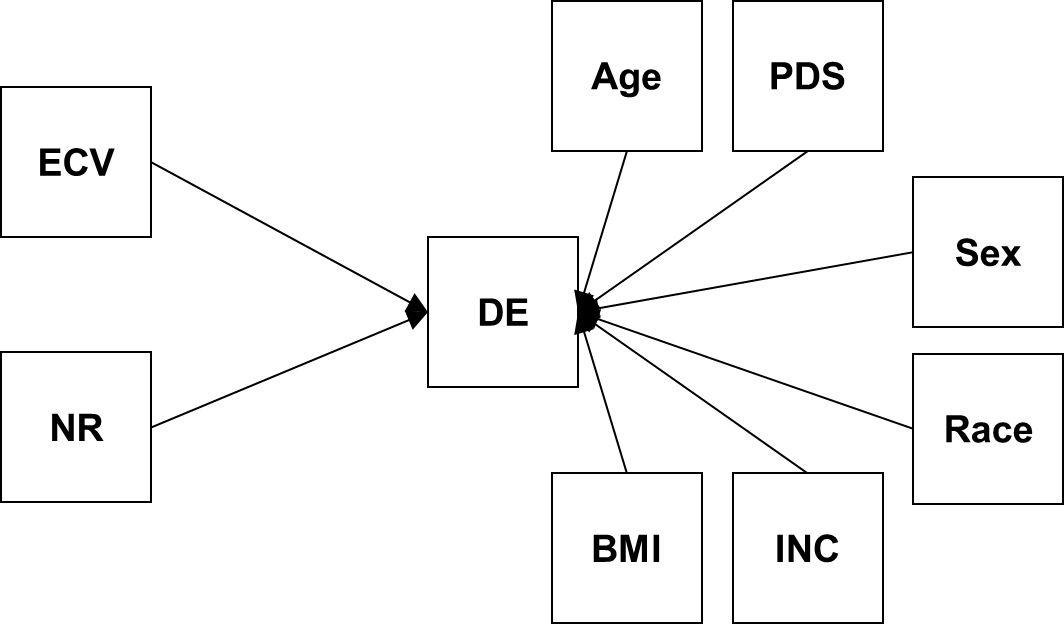
**

**Figure S1.** *Structural equation model for effects of community violence exposure and neighborhood resources on disordered eating.* DE = Minnesota Eating Behavior Survey total score; ECV = exposure to community violence; NR = neighborhood resources; PDS = Pubertal Development Scale score; INC = family income; BMI = body mass index percentile. Standardized and unstandardized path estimates are included in Table 3.

**
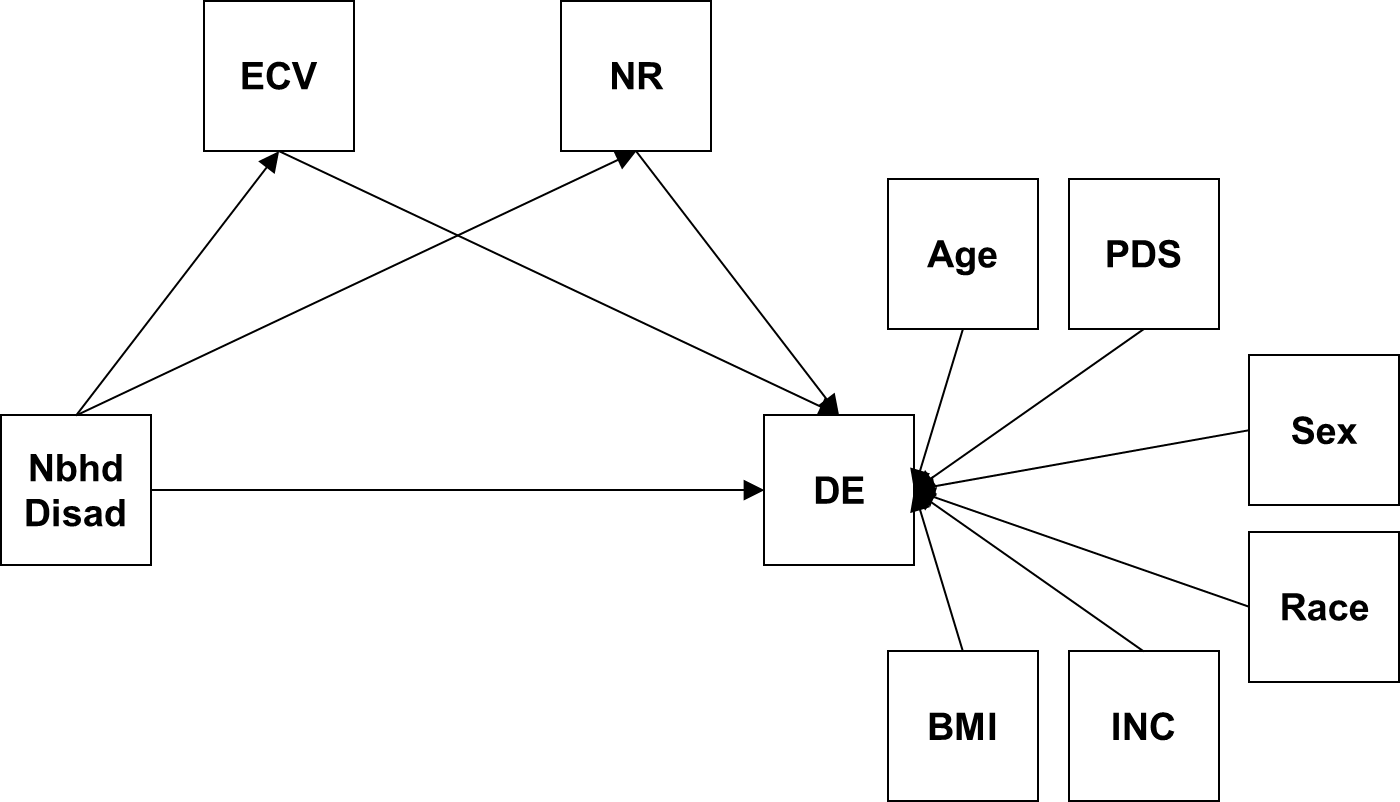
**

**Figure S2.** *Structural equation model for indirect effects of global neighborhood disadvantage on disordered eating through community violence exposure and neighborhood resources.* DE = Minnesota Eating Behavior Survey total score; ECV = exposure to community violence; NR = neighborhood resources; Nbhd Disad = global neighborhood disadvantage; PDS = Pubertal Development Scale score; INC = family income; BMI = body mass index percentile. Standardized and unstandardized path estimates are included in Table S3.

**
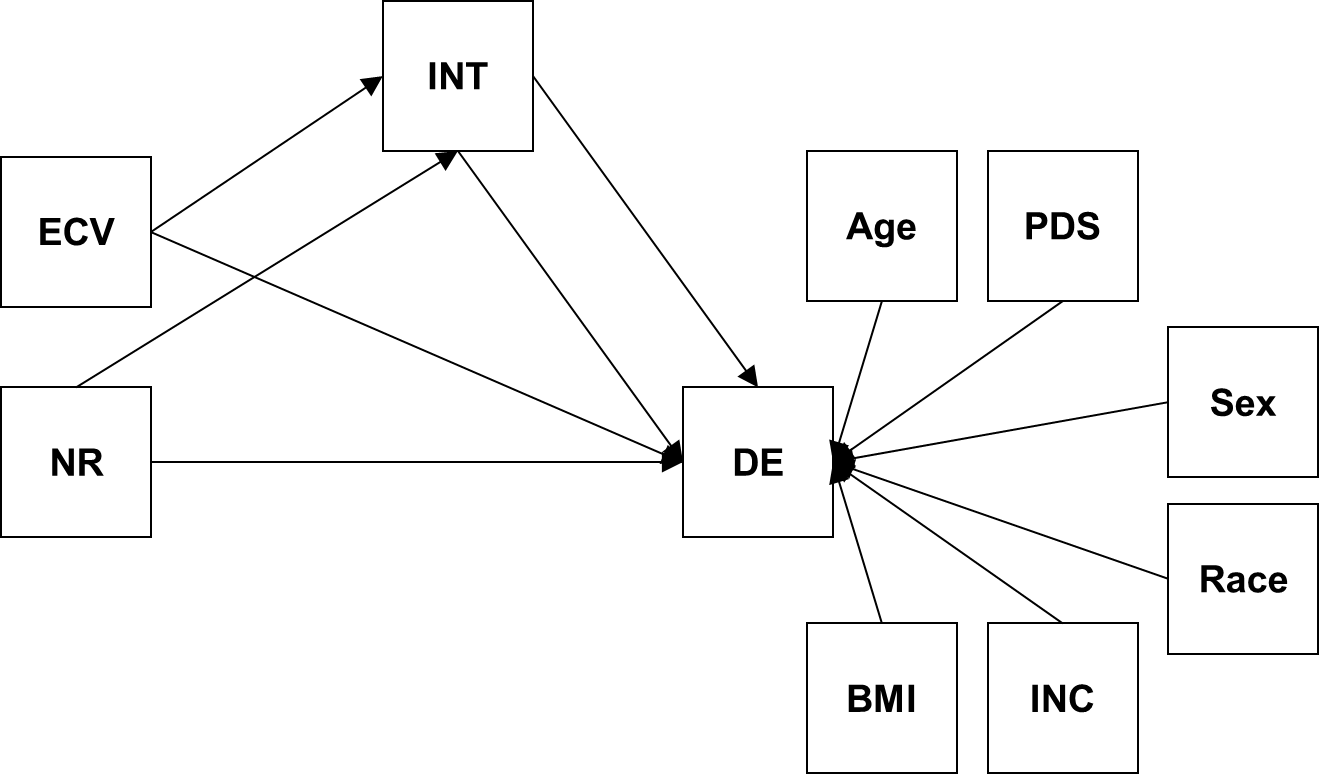
**

**Figure S3.** *Structural equation model for indirect effects of community violence exposure and neighborhood resources on disordered eating through internalizing symptoms.* DE = Minnesota Eating Behavior Survey total score; ECV = exposure to community violence; NR = neighborhood resources; INT = Structured Clinical Interview for Children and Adolescents internalizing scale score; PDS = Pubertal Development Scale score; INC = family income; BMI = body mass index percentile. Standardized and unstandardized path estimates are included in Table S4.
